# Genetic modification of human mesenchymal stem cells helps to reduce adiposity and improve glucose tolerance in an obese diabetic mouse model

**DOI:** 10.1186/s13287-015-0224-9

**Published:** 2015-12-09

**Authors:** Sabyasachi Sen, Cleyton C. Domingues, Carol Rouphael, Cyril Chou, Chul Kim, Nagendra Yadava

**Affiliations:** Department of Medicine, Division of Endocrinology and Metabolism, The George Washington University, School of Medicine and Health Sciences, 2300 I Street, Ross Hall Suite: 450, Washington, DC 20037 USA; Pioneer Valley Life Sciences Institute, and Division of Endocrinology, Diabetes & Metabolism at Baystate Medical Center of Tufts University School of Springfield, Springfield, MA USA

**Keywords:** Mesenchymal stem cells, SOD2, Superoxide, Diabetes, Glucose

## Abstract

**Introduction:**

Human mesenchymal stem cells (MSCs) are multipotent cells that can differentiate into fat, muscle, bone and cartilage cells. Exposure of subcutaneous abdominal adipose tissue derived AD-MSCs to high glucose (HG) leads to superoxide accumulation and up-regulation of inflammatory molecules. Our aim was to inquire how HG exposure affects MSCs differentiation and whether the mechanism is reversible.

**Methods:**

We exposed human adipose tissue derived MSCs to HG (25 mM) and compared it to normal glucose (NG, 5.5 mM) exposed cells at 7, 10 and 14 days. We examined mitochondrial superoxide accumulation (Mitosox-Red), cellular oxygen consumption rate (OCR, Seahorse) and gene expression.

**Results:**

HG increased reactive superoxide (ROS) accumulation noted by day 7 both in cytosol and mitochondria. The OCR between the NG and HG exposed groups however did not change until 10 days at which point OCR of HG exposed cells were reduced significantly. We noted that HG exposure upregulated mRNA expression of adipogenic (*PPARG, FABP-4, CREBP alpha and beta*), inflammatory (*IL-6 and TNF alpha*) and antioxidant (*SOD2 and Catalase*) genes. Next, we used AdSOD2 to upregulate SOD2 prior to HG exposure and thereby noted reduction in superoxide generation. SOD2 upregulation helped reduce mRNA over-expression of *PPARG, FABP-4, IL-6 and TNFα*. In a series of separate experiments, we delivered the eGFP and SOD2 upregulated MSCs (5 days post ex-vivo transduction) and saline intra-peritoneally (IP) to obese diabetic (db/db) mice. We confirmed homing-in of eGFP labeled MSCs, delivered IP, to different inflamed fat pockets, particularly omental fat. Mice receiving SOD2-MSCs showed progressive reduction in body weight and improved glucose tolerance (GTT) at 4 weeks, post MSCs transplantation compared to the GFP-MSC group (control).

**Conclusions:**

High glucose evokes superoxide generation, OCR reduction and adipogenic differentiation. Mitochondrial superoxide dismutase upregulation quenches excess superoxide and reduces adipocyte inflammation. Delivery of superoxide dismutase (SOD2) using MSCs as a gene delivery vehicle reduces inflammation and improves glucose tolerance in vivo. Suppression of superoxide production and adipocyte inflammation using mitochondrial superoxide dismutase may be a novel and safe therapeutic tool to combat hyperglycemia mediated effects.

## Introduction

Obesity and diabetes are increasing at alarming rates throughout the world leading to an increase in co-morbidities [1]. Dyslipidemia and insulin resistance predispose to type 2 diabetes and metabolic syndrome [2–4]. In this scenario, study of the effects of hyperglycemia and metabolic syndrome on adult stem cells or progenitors to mature endothelial cells, fat/muscle/cartilage cells is important. In this article we have focused on studying the effects of high glucose on long term structural stem cells, such as mesenchymal stem cells (MSCs). MSCs are multipotent stem cells that are precursors of osteoblasts, chondroblasts, and preadipocytes. They can differentiate into bone, cartilage, and adipocytes in the presence of a particular environment or cell culture condition [5]. They can be obtained from multiple sources, such as subcutaneous fat and bone marrow.

Increased oxidative stress and inflammation have been observed in adipose tissue pockets in hyperglycemia, diabetes, and obesity models [6–8]. It has been noted that human MSCs derived from muscle source when cultured in high glucose (25 mM glucose) and compared to normal glucose (5 mM glucose) promote accumulation of reactive oxygen species (ROS) [7–15]. However, a systematic and time dependent study of glucose effects on this important cell type is lacking.

In this study, we report the association between ROS and hyperglycemia, and the effect on adipogenic genes and cellular inflammation in human mesenchymal stem cells (MSCs). Most of our experiments used primary adipose tissue derived MSCs (ADMSC) and we also confirmed some of our cell based experiments using primary human bone marrow derived MSCs(BMMSCs).

### The biological paradox of oxygen and obesity

ROS are produced during normal cellular metabolism from incomplete reduction of oxygen. They have been shown to be cytotoxic, but also function as signaling molecules [7, 8, 10]. Therefore, understanding the mechanisms and pathways that regulate ROS homeostasis is crucial for the integrity of living organisms. This apparent paradox of beneficial vs cytotoxic effects of ROS can be balanced by well-developed enzymatic and non-enzymatic antioxidant systems to minimize the cellular damage and triggering of unwanted differentiation pathways caused by excessive ROS [11–13]. When ROS generation is increased to an extent that overwhelms the intracellular antioxidant defense systems, it leads to oxidative stress. This causes cellular and tissue damage by readily oxidizing critical macromolecules including proteins, lipids and nucleic acids, thereby contributing to the pathogenesis of many degenerative and/or chronic metabolic diseases. Recent literature suggests that increased ROS production from adipose tissue in obese subjects leads to systemic oxidative stress, primarily due to reduced antioxidant capacity, contributing to the development of obesity-linked diseases [14–17]. In simple terms, the antioxidant process progresses as follows: *nascent oxygen [O] + H*_*2*_*O ----via Superoxide Dismutase (either SOD1 or SOD2) converts to H*_*2*_*O*_*2*_*. H*_*2*_*O*_*2*_*is subsequently broken down to H*_*2*_*O and O*_*2*_*with the help of enzymes, i.e., catalase and glutathione peroxidase* (*GPX*). MnSOD or SOD2 is found in mitochondria and Cu-Zn SOD(SOD1), catalase and GPX are cytosolic [17].

## Methods

### Cell based assays and mitochondrial function assays

#### Cell culture and adipogenic differentiation

**Fig. 1:**
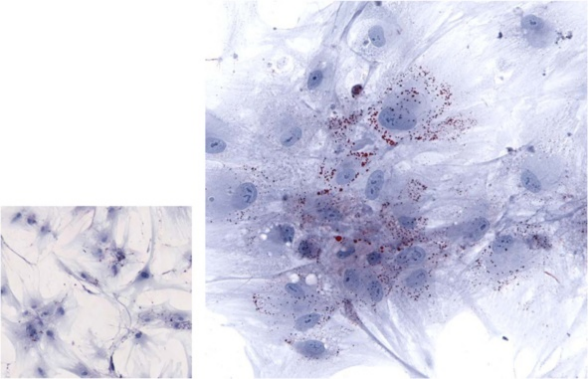
MSCs in normal and high glucose: DMEM-normal glucose on the *left* and in DMEM- high glucose on the *right*. The MSCs in high glucose show multiple fat droplet accumulation, intra-cellularly, stained with oil red O stain. Magnification: 40×. MSCs mesenchymal stem cells, *DMEM* Dulbecco’s modified Eagle’s medium

ADMSCs and BMMSCs were obtained from Lonza Walkersville, MD, USA. ADMSCs were initially cultured in DMEM normal glucose (NG, 5.5 mM; 1gm/L, Life Technologies Carlsbad, CA) containing 10 % fetal bovine serum and 1 % penicillin/streptomycin. Alternatively, cells were also cultured in the presence of DMEM high glucose (HG, 25 mM, 4.5gms/L, Life Technologies) up to 14 days. Culture medium was changed every 48 to 72 h until the cells were harvested. To induce adipogenic differentiation the cells were cultured in cycles of three days by alternating between adipogenic (NG or HG supplemented with 0.5 μM dexamethasone, 0.5 μM isobutylmethylxanthine, and 50 μM indomethacin) and maintenance medium (NG or HG). Fat droplets accumulation was assessed by Oil Red O staining [7, 8] Fig. 1. Culture conditions using NG or HG media were also referred to as normoglycemic or hyperglycemic respectively.

For BMMSCs we used MSCGM media from Lonza and adjusted glucose content to make it 25 mM for high glucose experiments.

#### RNA extraction, cDNA synthesis and gene expression

**Fig. 2:**
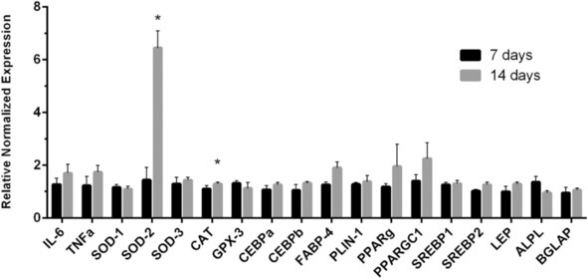
Effect of high glucose on MSC gene expression at Day 7 and 14. MSCs were exposed to normal and high glucose for 7 (*black bars*) and 14 (*gray bars*) days. Inflammatory, antioxidant, and adipogenic markers mRNA expression was analyzed by real time PCR. mRNA expression results of HG vs NG exposed cells were compared at the two different time points. Data are expressed as mean of three independents experiments ± SEM (paired t-test, *P < 0.05). *MSC* mesenchymal stem cell, *PCR* polymerase chain reaction, *HG* high glucose, *NG* normal glucose

**Fig. 3:**
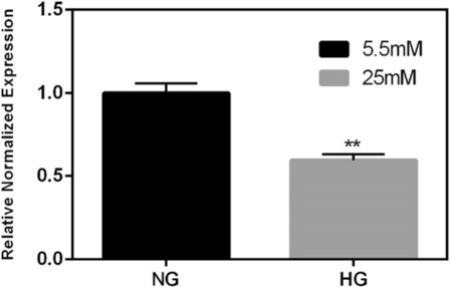
mRNA gene expression of Complex I- NDUFA1 (subunit of Complex I) of NG and HG cultured ADMSCs at day 14. NDUFA1 mRNA expression is statistically significantly lower in HG exposed cells NG normal glucose, *HG* high glucose, *ADMSCs* adipose tissue derived mesenchymal stem cells

Gene expression was assessed via reverse transcriptase PCR (RT-PCR). Total mRNA from adiposetissue-derived MSCs was extracted and purified using the RNeasy Mini Kit (Qiagen Germantown, MD). RNA conversion into cDNA was performed in T100 Thermal Cycler (Bio-Rad Hercules, CA) using the High Capacity cDNA Reverse Transcription Kit (Applied Biosystems Foster City, CA). The genes of interest were analyzed using CFX96 Real-Time System (Bio-Rad) in a 25 μL qPCR TaqMan reaction (Applied Biosystems). The expression of individual genes was normalized to either *18S* and/or *GAPDH* and analyzed as cycle threshold (Ct) difference. The target gene expression results are reported as ∆∆Ct values. Human genes tested in NG and HG, Figs. 2 and 3 were:* IL-6 (interleukin 6), TNFα (tumor necrosis factor- alpha), SOD1, 2 and 3 (superoxide dismutases), CAT (catalase), GPX-3 (glutathione peroxidase-3), CEBPa (cholesterol ester binding protein alpha), CEBPb (cholesterol ester binding protein beta), FABP4 (fatty acid binding protein-4), PLIN (perilipin), PPARG (peroxisome proliferator-activated receptor gamma), PPARGC1A (peroxisome proliferator-activated receptor gamma, coactivator 1 Alpha), SREBP1 (sterol regulatory element-binding protein 1), SREBP2 (sterol regulatory element-binding protein 2), LEP (leptin), ALPL (alkaline phosphatase) and BGLAP (osteocalcin) NDUFA1 (NADH:ubiquinone oxidoreductase subunit A1) and SDHB (Succinate Dehydrogenase Complex, Subunit B)*.

#### Complex I assay by BN-PAGE

**Fig. 4:**
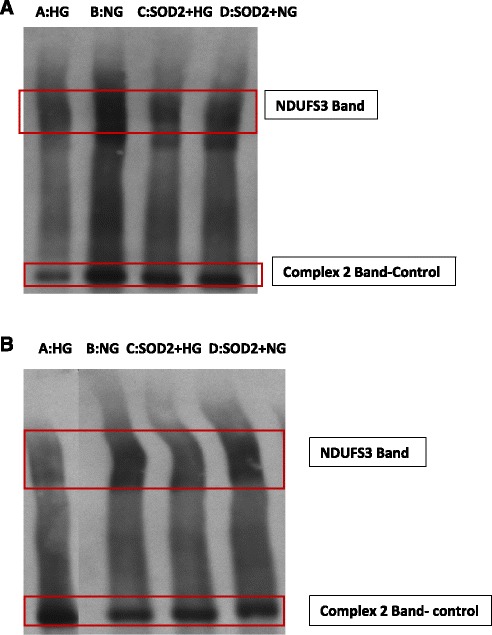
**a** Adipose tissue derived MSC (ADMSCs) cells in high glucose (HG) show mitochondrial Complex 1 (NDUFS3) dysfunction compared to normal glucose (NG). Complex 2 used as a loading control. A: high glucose exposed, B: normal glucose exposed, C: cultured in HG media following SOD2 upregulation, D: cultured in NG media following SOD2 upregulation. On comparing *Panel A* (HG, no SOD2) and *panel C* (HG with SOD2) we demonstrate that SOD2 up-regulation prior to HG exposure rescues NDUFS3 band. **b** Bone marrow derived MSC cells in HG show mitochondrial Complex 1 (NDUFS3) dysfunction compared to NG. Complex 2 used as a loading control. A: High glucose exposed, B: Normal glucose exposed, C: cultured in HG media following SOD2 upregulation, D: cultured in NG media following SOD2 upregulation. On comparing *Panel A* (HG, no SOD2) and *panel C* (HG with SOD2) we demonstrate that SOD2 up-regulation prior to HG exposure rescues NDUFS3 band. *SOD2* superoxide dismutase

Mitochondria from MSCs grown under NG or HG conditions were isolated by differential centrifugation and solubilized with dodecyl-β-D-maltoside (Sigma St Loius, MO) in 50 mM imidazole-HCl (pH 7.0) containing 5 mM 6-aminohexanoic acid and 10 % glycerol at 2:1 detergent: protein ratio. Oxidative phosphorylation (OXPHOS) complexes were separated by blue native-polyacrylamide gel electrophoresis (BN-PAGE) and detected using specific antibodies by Western blotting as described earlier [18, 19]. Respectively, Complexes I and II were detected using specific antibodies against NDUFS3 and SDHB (MitoSciences-Abcam Cambridge, MA) (Fig. 4a and 4b).

#### Oxygen consumption rate (OCR) determination

OCR was determined using Extracellular Flux Analyzers XFp and XF24-3 (Seahorse Biosciences Massachusetts, USA) as described [20, 21].

**Fig. 5:**
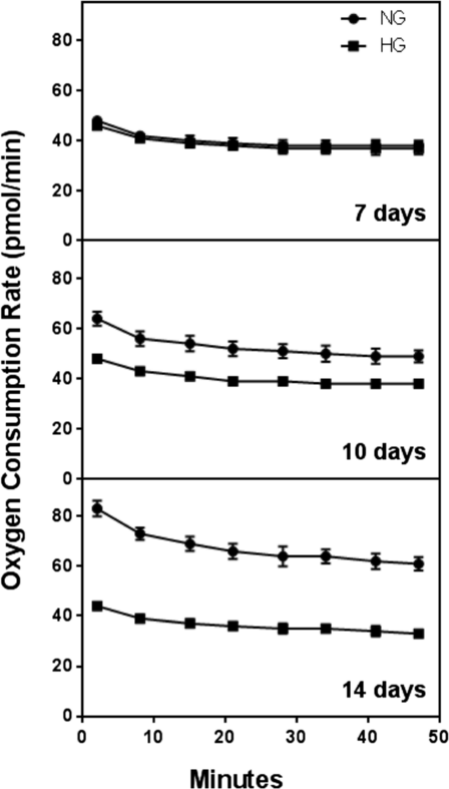
Comparison of MSC basal respiration in the presence of normal glucose (NG-*circles*) and high glucose (HG-*squares*). No cell permeabilizer was added. The results are representative and cells were cultured during 7 (*top*), 10 (*middle*), and 14 (*bottom*) days. *MSC* mesenchymal stem cells

For assays with XFp (in both Figs. 5 and 13) approximately 2 × 10^4^ per well were seeded in V7-PS mini-plates and allowed to grow in a CO_2_ incubator at 37 °C. After 24 hours cells were washed twice with assay medium (non-buffered XF Base Medium minimum DMEM; Seahorse Bioscience, Catalog: 102353–100), pH 7.4, containing 5.5 mM glucose, 1 mM pyruvate, and 2 mM glutamine and kept in the same medium. After that, cells were incubated in a CO_2_-free incubator at 37 °C for 30–60 min prior to the assay.

**Fig. 6:**
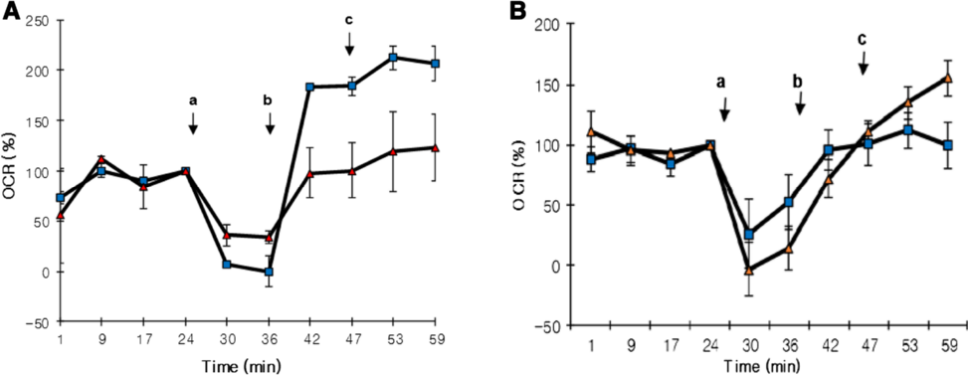
**a** and **b** Bone marrow derived MSC showing oxygen consumption rate (OCR) in percentage in normal glucose (NG, *squares*) and high glucose (HG, *triangles*) using *SeaHorse.* **a** shows better OCR in MSCs cultured in NG compared to MSCs cultured in HG, at day 7 of culture. Additives marked with *arrows*, A: 1nM rPFO, B: 10 mM glutamate and malate +2uM FCCP (Complex-I substrate), **c** 10 mM succinate (Complex-II substrate). **b** the experiment has been repeated with prior SOD2 upregulation in HG exposed cells, showing SOD2 upregulation did improve mitochondrial respiration, compared to the panel on *left*

We also used SeaHorse XF24, (Fig. 6) the MSCs were treated for seven days in NG and HG in their usual culture media. On the eighth day, 70000 of either normal or high glucose treated cells were seeded on the polyethyleneimine (PEI) coated XF24 well in appropriate media. The plate was placed in a hood without light for 30 min and was subjected to centrifugation for 10 min at 2,000 rpm at room temperature. The plate was incubated in a CO_2_-incubator for three hours. The plate medium was discarded and then the plate was washed with Ca2+-free low K+ buffer (LKB) containing 3.5 mM KCl, 120 mM NaCl, 0.4 mM KH2PO4, 1.2 mM Na2SO4, 2 mM MgCl2, 1mM EGTA (ethyleneglycol-tetra-acetic acid), 20 mM Na-N-Tris-(hydroxymethyl)-methyl-2-amino-ethanesulfonic acid (TES, pH7.4) and 0.4 % fat-free bovine serum albumin (BSA) unless otherwise noted. Respiration media were supplemented with glucose. Media was prepared and programs used for measurement as described before [20, 21]. The respiration of cells was measured with XF24 analyzer. The assay used 1 nM of rPFO (plasma membrane permeabilizer), 10 mM of glutamate, malate, and succinate, and 2 μM of FCCP. Before rPFO injection, two min mixing, two min waiting, and three min measuring and after rPFO injection, 30 sec mixing,1 min 30 sec waiting, and three min measuring cycles were used.

### Oxidative stress detection

**Fig. 7:**
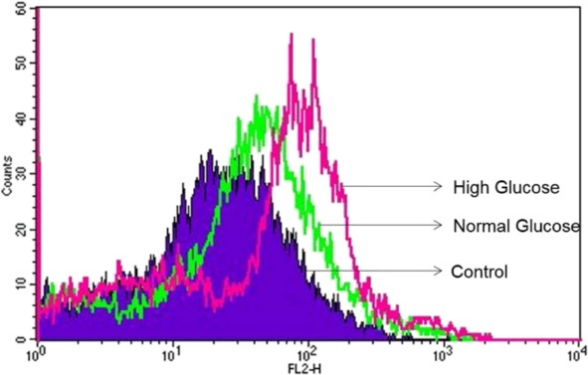
Mitosox Red Dye to detect ROS in MSCs in HG at day-7 of culture: Using FACS Analyzer increased mitochondrial fluorescence was noted. This increased fluorescence may be due to increased mitochondrial swelling, possibly secondary to ROS in high glucose state (red vs green). No difference was noted at Day1 or Day4 exposure to HG. Blue- unstained cells in NG (negative control), Green- MSCs in normal glucose and Red- MSCs in high glucose. *ROS* reactive oxygen species, MSCs mesenchymal stem cells, *HG* high glucose, *FACS* fluorescence activated cell sorting

**Fig. 8:**
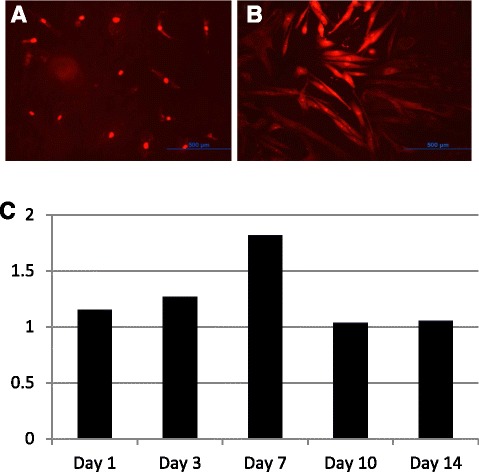
**a** (*left panel*) and **b** (*right panel*): Mitosox Red dye assay for detection and quantification of mitochondria generated reactive oxygen species presence intra-cellularly: Post culture at day 7 in NG and HG. *Left panel*: ADMSCs plated in normal glucose (NG, 5.5 mM), *Right panel*: ADMSCs plated in high glucose (HG, 25 mM). The fluorescence peaked at day 7 of culture and decayed beyond 14 days. Magnification: 10×. **c** Showing relative MitoSox fluorescence of normal glucose vs high glucose exposed cells over two weeks*. NG* normal glucose, *HG* high glucose, *ADMSCs* adipose tissue derived mesenchymal stem cells

Human adipose-derived MSCs (Lonza) were exposed to high glucose media (DMEM, 25 mM glucose) for a total of 14 days. At each of days 1, 3, 7, 10, and 14, cells were analyzed for superoxide accumulation inside the mitochondria using MitoSox red dye (Life Technologies), which is oxidized by ROS specifically inside the mitochondria. MitoSox was dissolved in DMSO to prepare a 5 mM stock solution. Stock solution was then dissolved in HBSS/Ca/Mg buffer to obtain a 5 μM working solution. Cells were cultured on a 24-well plate (25,000cells/ well) Fig. 7. Wells were stained at each of days 1, 3, 7, 10 and 14 with 5 μM MitoSox solution and fluorescence was measured at 530/590 nm by a plate reader, Fig. 8.

hMSCs cultured in HG media were stained again with MitoSox and then observed under the microscope at each of days 2 and 7 to check for fluorescence. The same experiment was repeated using Mitotracker Green FM (Life Technologies) dye instead of MitoSox and fluorescence was checked under the microscope at day 7 to assess mitochondrial mass.

**Fig. 9:**
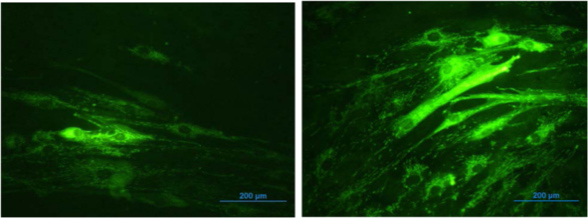
Mitotraker Green Dye Assay in NG and HG: NG (*Left*) and HG *(right*): Helps to visualize mitochondrial mass in HG, which unlike NG shows fragmented fibrillary structures that are uniformly more fluorescent, at day 7 of culture; magnification: 20×. *NG* normal glucose, *HG* high glucose

**Fig. 10:**
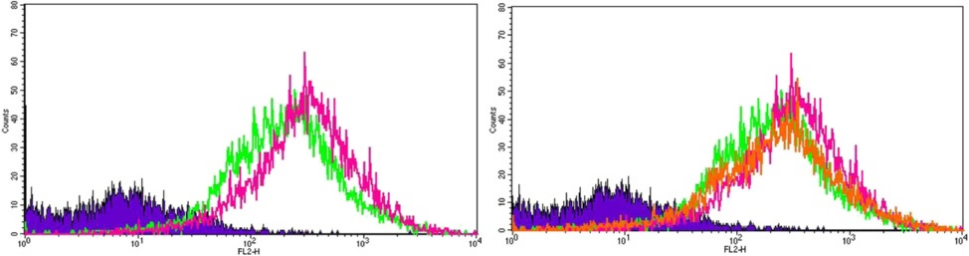
Mitosox Red Dye to detect ROS in MSCs in HG at Day-14 of culture, using FACS Analyzer: shows increased mitochondrial swelling, possibly secondary to ROS in high glucose state (red vs green). Blue- SCM (negative control), green- MSCs in normal glucose and red- MSCs in High Glucose, Orange: MSCs transduced with AdSOD2 and then exposed to high glucose. The figure on the *right* indicates that MSCs post SOD2 up-regulation (orange) have less fluorescence than without (*red line*). In both cases the MSCs were HG exposed. *ROS* reactive oxygen species, *MSCs* mesenchymal stem cells, *FACS* fluorescence activated cell sorting, *SOD2* superoxide dismutase

For the Mitotraker experiment Fig. 9, we plated 25,000cells/chamber on a two chamber slide. Two chambers had cells cultured in NG media and two chambers had cells cultured in HG media. At day 7, cells were incubated in Mitotracker working solution (200nM) for 30 minutes, washed with PBS and observed under the microscope using the red filter (Ex:490/Em: 516 nm) for fluorescence. In Fig. 10, MitoSox experiment was repeated at day 14 with NG and HG exposed cells along with SOD2 upregulated cells, to note if effects of HG can be aborgated.

### Cell Based Assays post superoxide dismutase (SOD) gene upregulation

**Fig. 11:**
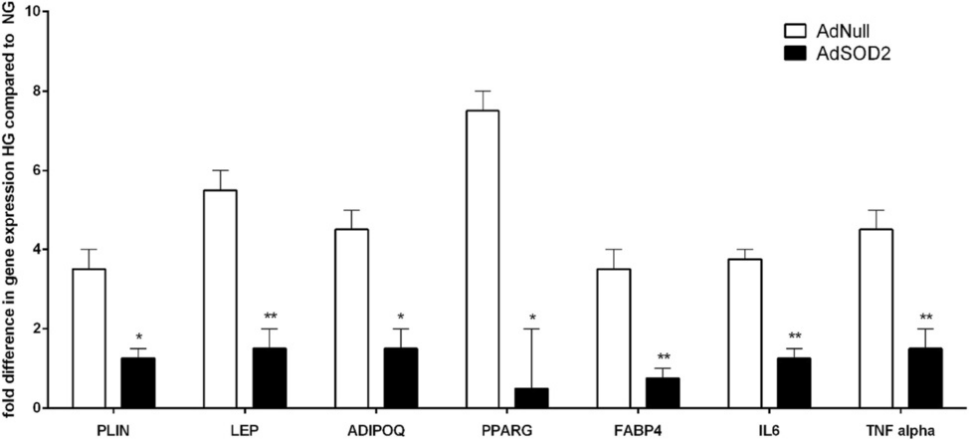
Gene expression of ADMSCs in high glucose, post transduction with Null or SOD2. ADMSC on culture for 14 days also showed reduction in expression of both fat/adipogenic genes and also inflammatory genes (Fig. 11) when compared to Fig. 2. MSC mesenchymal stem cells, SOD2 superoxide dismutase. *ADMSCs* adipose tissue derived mesenchymal stem cells

**Fig. 12:**
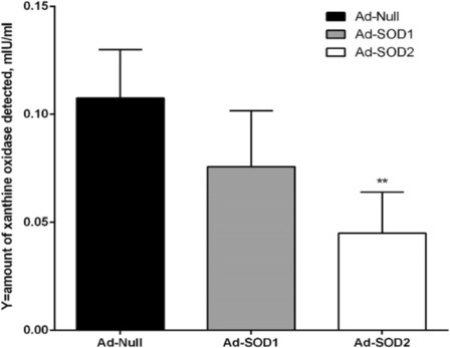
Intracellular SOD assay: detection of SOD activity in SOD1 and SOD2 upregulated cells compared to AdNull transduced cells. *SOD* superoxide dismutase. To ensure that superoxide dismutase (SOD) has been truly upregulated intracellularly we assessed SOD activity (Fig. 12) and demonstrated that SOD2 upregulated MSCs have more dismutase activity and used less xanthine oxidase compared to SOD1 upregulated cells indicating that SOD2 upregulated cells have more SOD activity than SOD1. Xanthine oxidase (XO) is a key enzyme necessary to produce superoxide. AdNull transduced cells were associated with maximum XO, as expected, and thereby was associated with least SOD activity

**Fig. 13:**
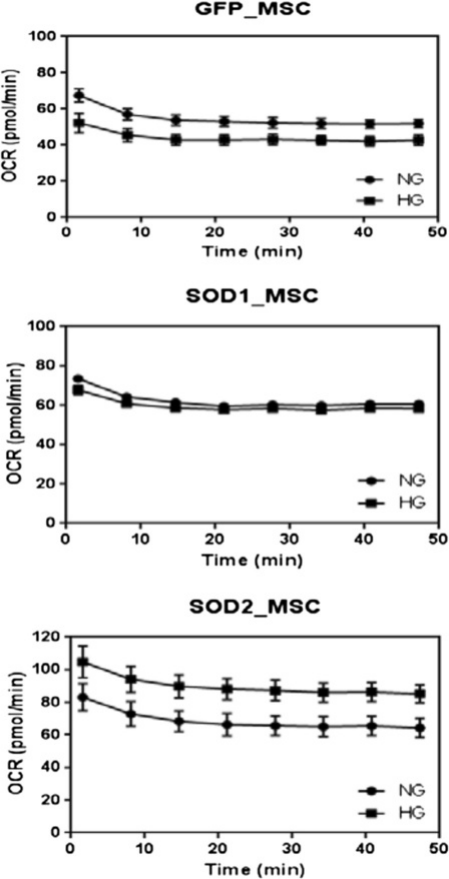
Comparison of ADMSC respiration in the presence of normal glucose (NG-*circles*) and high glucose (HG-*squares*), following transduction with GFP, SOD1 and SOD2 at day 10 of NG or HG culture. *ADMSC* adipose tissue derived mesenchymal stem cells, *GFP* green fluorescent protein, *SOD* superoxide dismutase

Figure 11 shows RT-PCR of adipogenic and pro-inflammatory genes pre and post SOD2 over-expression in HG. SOD2 upregulation reduces high expression of these genes in HG. Figure 12 shows SOD activity assay (Xanthine Oxidase Assay) in Ad Null (control), AdSOD1 and AdSOD2 upregulated ADMSCs, detailed below. Figure 13 shows OCR tracings using Seahorse XFp machine, of ADMSCs, in NG and HG post GFP (control), SOD1 and SOD2 upregulation at day 10.

### Xanthine oxidase fluorometric assay

We used a Superoxide Dismutase Assay Kit from Sigma Aldrich (#19160) to detect xanthine oxidase and quantify superoxide dismutase activity intracellularly. We tested AdSOD1, AdSOD2, and AdNull (all constructs obtained from Vector Biolabs Malvern, PA, USA) transduced cells and measured intra-cellular SOD activity. In this assay superoxide anions (O_2_^−^) are generated by a xanthine/xanthine oxidase (XOD) system, and then detected with a chromagen solution. However, in the presence of SOD, these superoxide anion concentrations are reduced, yielding less colorimetric signal (Fig. 12).

### Animal glucose tolerance tests and body composition assessment

Animals used for this experiment were db/db leptin resistant obese diabetic mice obtained from Jackson Labs, Bar Harbor, ME, USA (Jackson Lab, Cat # 642). All animals prior to intraperitoneal (IP) MSC delivery had tail blood glucose levels above 250 mg %, tested by one-touch ultra glucometer.

Glucose tolerance test: All mice used were fasted for 24 hours prior to testing. Glucose solution was synthesized using dextrose solid crystals from Sigma Aldrich (cat # D9434) with a general working solution of 0.5 g diluted in 1 mL of normal sterile saline from Hospira Lake Forest, IL (cat # 488810) and subsequent sterilization via a 0.2 μm pore filter into a vacutainer tube for intraperitoneal injection.

Intraperitoneal injections were performed by standard animal care technique. Mice were lifted by the scruff of their backs and dextrose solution was injected into the lower left intraperitoneal region using a 1 cc syringe and 25 gauge 5/8” needle. Dextrose delivery was calculated at a dose of 2 g per 1 kg body weight. Blood glucose of mice was measured using an Abbott Free-Style Lite (detection range 20-500 mg/dL) by tail tip vein needle puncture. Glucose measurements were made in increments of 0, 15, 30, 45, 60, 90, and 120 minutes, with time 0 being a glucose measurement recorded prior to intraperitoneal injection. Body composition (in grams – fat, lean mass, free water, and total water) was measured by MRI using an EchoMRI™-100H Analyzer [22]. Due to the nature of the hardware, all mice were alive during measurements and not given anesthesia. No particular preparations were made prior to, during, or after MRI measurements. Mice were assisted into fitted breathable and closable hollow plastic cylinders according to their weight where the cylinder was placed into the analyzer for MRI scanning which took place over the course of approximately three minutes. After measurements, mice were let out of their cylinders and assisted back to their respective cages. [EchoMRI:http://www.echomri.com/Body_Composition_Mice_2MHz.aspx].

The protocol was approved by Baystate Medical Center, Springfield, MA, USA, Institutional Animal Care and Use Committee (IACUC) and IBC Board [#134855-13 Antioxidant gene transfer using adenovirus] and all experiments were conducted under approved ethical guidelines. The protocol was also approved by IACUC and IBC, George Washington University, Washington DC, #A-335.

## Results

To determine the effect of hyperglycemia MSCs were cultured under HG and NG media for comparison. The levels of lipid droplets, ROS, mitochondrial function, and expressions of selected marker genes were assessed. Our data suggest that HG conditions increased lipid droplet accumulation (colored red, following Oil Red O staining) at day 14 of culture (Fig. 1).

To determine how exposure to HG affects adipogenic differentiation of MSCs, we measured expression of selected genes. Our data show that HG treated MSCs presented over-expression of fat genes PPAR-Gamma, FABP-4, inflammatory genes TNF- alpha, and IL6 with statistical significant upregulation of antioxidant genes, particularly SOD2 or MnSOD and catalase, at day 14 post exposure (Fig. 2).

We have also controlled for mannitol. Compared to Fig. 2 of our manuscript there was no difference in any genes for Day 7 and day 14 when cells exposed to NG was compared to NG+ equivalent mannitol. No cellular toxicity was noted at two weeks of HG exposure.

Figure 2 also demonstrates that bone marker, such as alkaline phosphatase gene (ALPL), mRNA expression is reduced over time in HG environment. *HG* high glucose.

The very high expression of SOD2 in HG treated cells could be a defensive strategy or a strategy to increase H_2_O_2_ production for signaling purposes. Elevated levels of mitochondrial ROS were also observed using MitoSOX probe by flow cytometry at day 7. A rightward shift in MitoSOX fluorescence in HG compared to NG is clearly indicative of increased mitochondrial superoxide levels (Fig. 7). Fluorescence microscopic analysis of MSCs also showed enhanced MitoSOX staining in MSCs cultured in HG (Fig. 8). A time course of MitoSOX fluorescence by plate-reader demonstrated peak fluorescence at day 7 post HG treatment (Fig. 8c). Mitotraker-Green also confirmed similar findings at day 7 post commencement of culture validating superoxide accumulation in mitochondria (Fig. 9). Since impaired respiratory chain function in hyperglycemia may result in oxidative stress, we measured relative mitochondrial function in hyperglycemia vs. normoglycemia, such as oxygen consumption rate (OCR) using Seahorse Bioscience Extracellular Flux Analyzers (XF24, XFp). The MSCs cultured in hyperglycemic conditions showed lower respiration rates using XFp (Fig. 5). The negative effect of HG on MSCs respiration increased with time. Figure 5 demonstrates the progressive OCR reduction over 7, 10 and 14 days after the start of MSCs culture in the HG condition. The reduced OCR seen in HG exposed cells could be due to reduced level of assembled respiratory chain Complex I (NADH-ubiquinone oxidoreductase). This was supported by reduced mRNA expression of NDUFA1, a Complex 1 subunit (Fig. 3), indicating possible Complex-I deficiency in hMSCs in the presence of high glucose. RTPCR of Complex II (SDHB) did not show any change in NG vs. HG exposed MSCs. Reduced Complex I level and function were further supported by respirometry and BN-PAGE data (Fig. 4). We tested the effect of SOD2 over expression using an adenoviral viral vector (AdSOD2) from Vector BioLabs. AdSOD2 over expression prior to HG exposure (orange line) appears to have reduced MitoSOX fluorescence previously seen in HG-treated cells (Fig. 10a and 10b). To assess whether oxidative stress could be associated with reduced respiration and Complex I deficiency, we assessed respiration and Complex I levels in MSCs treated with NG and HG in the presence and absence of AdSOD2. Figure 4a demonstrates lower Complex I activity is associated with low levels of assembled Complex I as revealed by BN-PAGE, in ADMSCs which were rescued by AdSOD2. Incidentally, the same finding is also noted in BMMSCs (Fig. 4b). The recovery of Complex I by AdSOD2 suggests the regulation of some aspects of Complex I biogenesis by ROS signaling.

Figure 6a demonstrates that in HG exposed bone marrow derived MSCs Complex I-dependent respiration was relatively less. The glutamate- plus malate-supported respiration was significantly less in HG compared to NG MSCs. However, the Complex II-dependent respiration supported by succinate as substrate was also lower in HG cells. Figure 6b shows that SOD2 up-regulation in MSCs prior to HG exposure did improve mitochondrial respiration, compared to the panel on left.

Figure 13 compares ADMSC respiration in the presence of normal glucose and high glucose, following transduction with adenovirus GFP (control-top panel), adenovirus SOD1 (middle panel) and adenovirus SOD2 (*bottom panel*) at day 10 of NG or HG culture. SOD2 transduced cells following HG exposure actually improved respiration even more than NG exposed cells. SOD1 also improved respiration but not to the extent of SOD2 transduced cells.

**Fig. 14:**
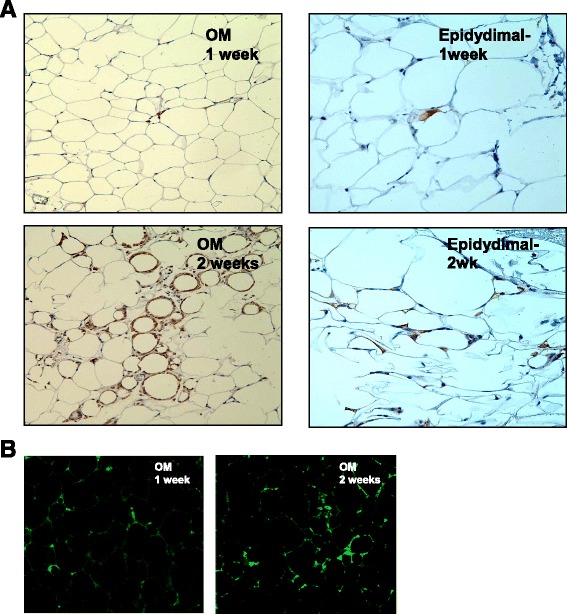
**a** GFP detection in fat depots at week 1 (*upper panel*) and week 2 (DAB secondary) with brown staining (*lower panel*). **b** GFP detection in omental fat depots at week 1 and week 2 by direct laser confocal microscopy is also shown. GFP green fluorescent protein,*DAB* 3,3'-diaminobenzidine

In order to develop a therapeutic aspect to our findings we decided to deliver SOD2 upregulated cells to reduce inflammation and insulin resistance secondary to inflammation. When we delivered 1.5 million MSCs (transduced with AdGFP) intraperitoneally, we demonstrated using the appropriate staining method that an adequate number of MSCs reached subcutaneous, omental and epididymal fat pockets (Fig. 14a and 14b). We have also verified by direct fluorescence that GFP upregulated MSCs had reached the fat pockets. The “homing-in” of MSCs to fat pockets is possibly secondary to inflammatory molecules produced from fat pockets and can be detected as early as one week post intraperitoneal (IP) delivery; however,. assimilation is significantly better by the second week of IP delivery, when MSCs may actually be coalescing with fat pocket host cells (Fig. 14a, week 2 OM and week 2 EP). Figure 14a shows GFP staining with DAB as secondary staining agent for omental and epididymal fat. Figure 14b demonstrates direct GFP staining for omental fat. The secondary staining method provided better clarity, confirming GFP positive cells reaching fat depot and subsequently assimilating in host tissue.

Next, we delivered hMSCs that were transduced prior to delivery intraperitoneally (IP) with AdSOD2, in mice to determine if over-expressing SOD2 in human MSCs can reduce local inflammation and insulin resistance. After “homing-in” these hMSCs possibly lie adjacent or assimilate into native adipocytes, mimicking a co-culture scenario. Five animals in each of three groups received IP administration of the pre-determined number of hMSCs transduced with: 1) AdGFP (control for adenoviral vector), 2) AdMnSOD suspended in 200 uL of PBS, and 3) saline. hMSCs were transduced ex vivo and cultured for five days before being delivered to db/db (Jackson Labs) mice IP.

**Fig. 15:**
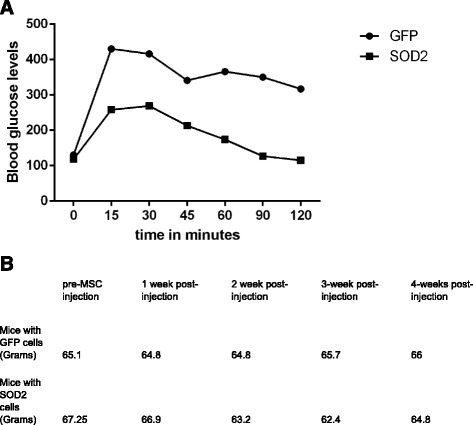
**a** Glucose tolerance test in db / db mice 28 days after MSC delivery, intraperitoneally. **b** Weight of mice over four weeks that received either GFP or SOD2 up-regulated MSCs*. MSC* mesenchymal stem cell, *GFP* green fluorescent protein*, SOD2* superoxide dismutase 2

Outcome measures were glucose tolerance tests and body weight and composition measurement. (Fig. 15a and 15b).

We demonstrated that transplantation of MnSOD up regulated MSC improved glucose tolerance (Fig. 15a) and total body weight and total fat mass in obese and diabetic db/db mice compared to mice receiving eGFP up-regulated MSCs, gradually reduced over the four week observation period (Fig. 15b). There was no statistically significant difference in body weight and body fat mass between mice receiving GFP transduced MSCs and saline over four weeks, post IP delivery of cells or saline.

## Discussion

In this study we investigated the effect of glucose (HG vs NG) on human subcutaneous adipose tissue derived-MSCs. In the recent past we have investigated the effect of exercise on diabetes and prediabetes patients using CD34+ progenitor cells [23]. A significant percentage of diabetes patients [24], particularly type 2, do present levels of 20-25 mM of glucose (360 mg % to 450 mg %) and, therefore, discerning the effect of high levels of glucose on undifferentiated multi-potent mesenchymal stem cells is very important. It is also important to discern the factors that influence MSCs differentiation in a particular fashion and whether one can manipulate gene expression ex-vivo so as to influence differentiation.

From our series of experiments, we noted that HG accentuates triacylglycerol accumulation in MSCs and this is associated with ROS presence particularly in mitochondria which seems to peak at day 7 post HG exposure (Fig. 8). The effect of ROS accumulation is also noted in reduced oxygen consumption rate (OCR) of these cells at day 10, which is accentuated at day 14. The reduced OCR seems to be secondary to OxPhos Complex I deficiency. While the lower expression of NDUFA1 mRNA and reduced Complex I content suggest that Complex I assembly may be impaired in high glucose, a higher turnover of Complex I cannot be excluded. No difference in Complex II content but its activity points toward functional dysregulation of Complex II. The correction of respiratory chain deficiency by SOD2 suggests that oxidative stress impairs the OXPHOS system in MSCs experiencing hyperglycemia.

Interestingly, the gene expression analysis showed that up to day 7 of HG exposure there seems to be no major difference between NG and HG exposed cells. However, at day 14 the two principal genes that seem to be affected are SOD2 and catalase both being anti-oxidants, of which SOD2 is several fold upregulated. This phenomenon is probably a cellular response to high glucose induced ROS. Like others we, too, noted an upregulation of adipogenic genes, such as FABP4 and PPARG, mature adipocyte marker genes, such as perilipin, leptin and adiponectin, and inflammatory genes, such as TNF alpha and IL6 [7].

Based on our finding from qPCR the critical genes which the HG exposed cell tries to upregulate itself as an attempt to self-preserve are anti-oxidants, particularly SOD2 (mitochondrial SOD or MnSOD) (Fig. 2). Therefore, to verify whether the effects induced by HG could be reversible, we supplied the SOD2 ex-vivo, using adenovirus-SOD2 by gene transfer. We decided to use adenovirus, a DNA virus, in order to over-express a specific gene inside the MSC [25, 26].

Post SOD2 upregulation the detrimental effects of HG on adipo-derived MSCs seem to be halted. The respiratory activity improved and Complex I dysfunction was reversed. Post SOD2 upregulation HG exposed cells demonstrate a reduction in adipogenic genes such FABP4 and PPARG, mature adipocyte marker genes such as perilipin, leptin and adiponectin and inflammatory genes such TNF alpha and IL6.

We wanted to insure that the antioxidant upregulated cells actually do have more superoxide dismutase and did an assay to show there was more dismutase intra-cellularly. Interestingly, SOD activity assay was more robust in SOD2 upregulated cells than SOD1 compared to AdNull upregulated cells. SOD activity was also noted in the conditioned media obtained from AdSOD1 and AdSOD2 transduced cells unlike AdNull transduced MSCs, indicating a beneficial paracrine property of the anti-oxidant upregulated cells. Similarly, SOD1 transduced cells showed better OCR than GFP transduced cells but SOD2 transduced cells showed the best respiration. Figures 4, 5 and 6 demonstrated that HG mediated Complex I defect is not specific to site of origin for the MSCs. In fact, both ADMSCs and BMMSCs demonstrate a similar effect of poor respiration and Complex I defect on exposure to HG beyond seven days.

To investigate therapeutic utility of genetically modified MSCs we delivered AdGFP upregulated adipo derived-MSCs intraperitoneally. We used ADMSCs because we hoped to show coalescing of MSCs with the host tissue and thereby deliver the gene and its paracrine properties, better, to the surrounding inflamed adipose tissues. We demonstrated that GFP up-regulated cells do reach the distal fat pockets possibly chemo-attracted by inflammatory cytokines being released from the fat depots in the db/db leptin resistant mice. Indeed, the most GFP positive cells (stained with GFP antibody and DAB secondary) seem to be evident in the omental fat rather than any other fat depots, followed by subcutaneous and epididymal fat. This finding is most probably due to the route of cell delivery, which is intra-peritoneal.

When we delivered SOD2 upregulated cells and compared with delivery of GFP upregulated cells we noted a gradual decrease in fat mass (using Echo-MRI) in the first group. At four weeks post-delivery of MSCs we performed a glucose tolerance test (GTT) which clearly showed improvement in GTT (n = 5) for the group that received SOD2 upregulated MSCs. This is possibly secondary to reduction in inflammation which reduced insulin resistance, fat mass and improved glucose utilization. In this project we used the “homing” property of the human MSCs which are known to home to the site of inflammation and injury to deliver anti-oxidant at a niche where ROS is produced. We also utilized the non-immunogenic property of the MSCs to deliver the cells in vivo in a mouse model of diabetes and obesity. This technique can be utilized as a novel and safe therapy in subjects suffering from obesity and diabetes, utilizing their own subcutaneous fat derived MSCs. Adenovirus used to up-regulate human antioxidants is a DNA virus and remains as an episomal character with no integration with host genome. It is therefore safe for ex-vivo transduction and gene upregulation before the virus free cell suspension is delivered intraperitoneally. A limitation to this process for human therapy in a chronic disease setting of diabetes and obesity will be short term expression of adenovirus mediated gene transfer, which would require multiple injections over a period of time. To avoid this issue, one can use adeno-associated virus mediated gene transfer [23, 25] without causing long term side-effects.

## Conclusions

Hyperglycemia accentuates fat formation and adipogenesis in undifferentiated mesenchymal stem cells which is associated with increased mitochondrial superoxide accumulation, cellular inflammation and impaired cellular respiration. The impaired cellular oxygen consumption and Complex 1 dysfunction is not specific to adipose derived MSCs but is also seen in BMMSCs. When excess mitochondrial superoxide is reduced using mitochondria specific SOD2 gene upregulation the hyperglycemia induced effects are abrogated including reduction of cellular inflammation. We decided to investigate further if these genetically modified MSCs can be utilized in an obese diabetic mouse model. We demonstrated that MSCs do home-in to inflamed adipocyte pockets and SOD2 upregulated cells on delivery in diabetic obese mice do show reduction in weight with improved glucose tolerance tests compared to control. This finding can have significant impact in diabetes therapeutics using human MSCs, which could be obtained commercially or obtained from diabetic subject’s fat depots.

## Abbreviations

ADMSC: Adipose tissue derived mesenchymal stem cells
BMMSC: Bone marrow derived mesenchymal stem cells
GTT: Glucose tolerance test (intraperitoneal)
HG: High glucose (25 mM)
MSC: Mesenchymal stem cells
NG: Normal glucose (5.5 mM)
OCR: Oxygen consumption rate
ROS: Reactive oxygen species
SOD: Superoxide dismutase enzyme
